# Appendiceal neurofibroma with low-grade appendiceal mucinous neoplasm in neurofibromatosis type 1 patient: A case report

**DOI:** 10.1016/j.ijscr.2018.11.005

**Published:** 2018-11-13

**Authors:** Toshiaki Komo, Koichi Oishi, Toshihiko Kohashi, Jun Hihara, Masanori Yoshimitsu, Noriaki Tokumoto, Mikihiro Kanou, Akira Nakashima, Yoshirou Aoki, Manabu Shimomura, Masashi Miguchi, Mahito Funakoshi, Hidenori Mukaida, Mayumi Kaneko, Hiroo Matuura, Naoki Hirabayashi

**Affiliations:** a Department of Gastroenterological Surgery, Hiroshima City Asa Citizens Hospital, Japan; b Department of Pathology, Hiroshima City Asa Citizens Hospital, Japan; c Department of Gastroenterological and Transplant Surgery, Applied Life Sciences, Institute Biomedical & Health Sciences, Hiroshima University, Japan

**Keywords:** NF1, neurofibromatosis type 1, LAMN, low-grade appendiceal mucinous neoplasm, CT, computed tomography, MPNST, malignant peripheral nerve sheath tumor, Neurofibromatosis type 1, von Recklinghausen’s disease, Appendiceal neurofibroma, Low-grade appendiceal mucinous neoplasm

## Abstract

**Introduction:**

Neurofibromas are a characteristic of the autosomal dominant disorder Neurofibromatosis type 1 (NF1), also known as von Recklinghausen’s disease. Appendiceal neurofibromas are extremely rare, and low-grade appendiceal mucinous neoplasms (LAMNs) have not previously been reported in NF1.

**Presentation of case:**

A 62-year-old man with NF1 was scheduled for elective surgical treatment of an asymptomatic, enlarged and diffusely thickened appendix that remained after curative antimicrobial treatment of acute appendicitis. Laboratory analysis revealed all normal. Colonoscopy showed thickened appendiceal mucosa projecting into the cecum. A sample of the mucosa was found to be pathologically benign. The patient was preoperatively diagnosed with treated acute appendicitis with chronic appendiceal inflammation versus appendiceal neoplasms. Laparoscopic cecectomy was performed. Multiple neurofibromas were observed in the muscle layer, submucosa, and mucosa of the appendix on histopathological examination. Immunohistochemical examination showed positive staining for S-100. Pathologically, the patient was diagnosed with appendiceal neurofibroma consistent with NF1 with LAMNs. His postoperative course was unremarkable. He was discharged on post-operative day 3 and remained in good health 7 month after surgery.

**Conclusions:**

Appendiceal neurofibromas are often preoperatively diagnosed as appendicitis. Appendiceal neurofibromas should be considered in patients with NF1 who are suspected of having appendicitis.

## Highlights

• Appendiceal neurofibromas (AN) in Neurofibromatosis type (NF) 1 are rare.
• AN in NF1 with Low-grade appendiceal mucinous neoplasms (LAMNs) are extremely rare.
• AN and LAMNs have potential for malignant transformation.
• Surgical resection is the standard treatment for patients with AN and LAMNs.
• However, appropriate surgical procedure remains controversial.

## 1 Introduction

Neurofibromatosis type 1 (NF1), also known as von Recklinghausen’s disease, is an inherited autosomal dominant neurocutaneous syndrome. NF1 is a multisystemic disorder that can affect any organ in the body. The most typical clinical presentations are neurofibromas and café-au-lait spots [1]. Neurofibromas are found in the gastrointestinal tract in 11% of patients with NF1 [2]. However, appendiceal neurofibromas in NF1 are extremely rare. Furthermore, low-grade appendiceal mucinous neoplasms (LAMNs), characterized by low-grade cytologic atypia and absence of destructive invasion [3], have not been reported in NF1.We report herein a case of appendiceal neurofibromas in NF1 with LAMNs. The present work has been reported in accordance with the SCARE criteria [4].

## 2 Case presentation

**Fig. 1:**
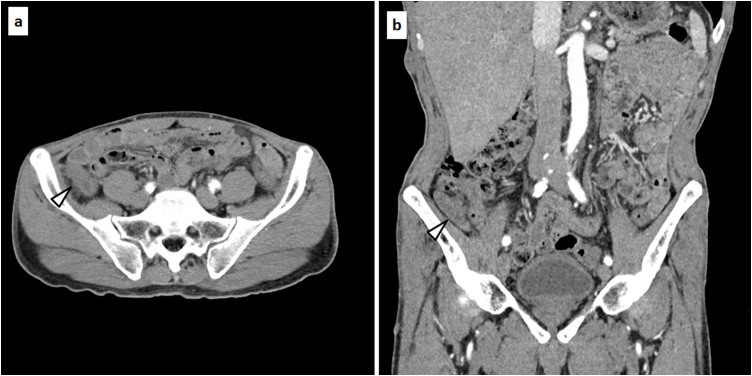
Contrast-enhanced CT showed an enlarged and diffusely thickened appendix (appendix: thick white arrow head).

**Fig. 2:**
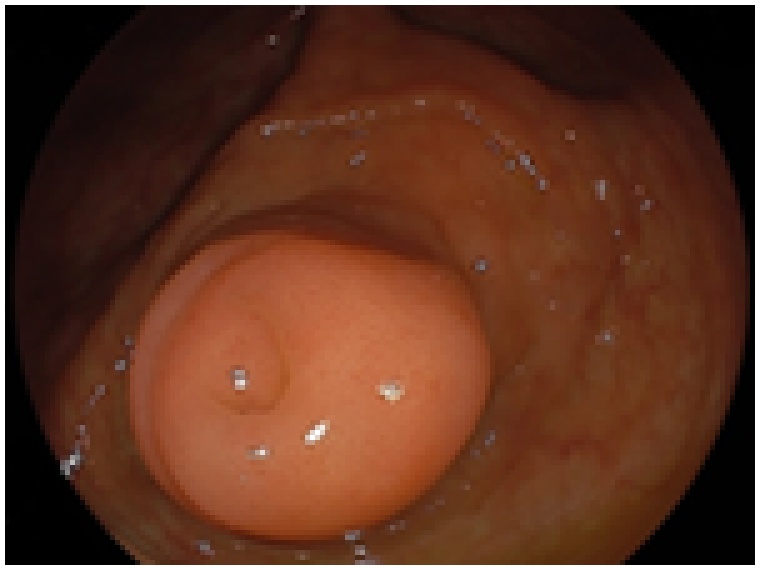
Colonoscopy showed a thickened mucosa of appendix projecting to the cecum without outflow of abscess or jellylike liquid.

**Fig. 3:**
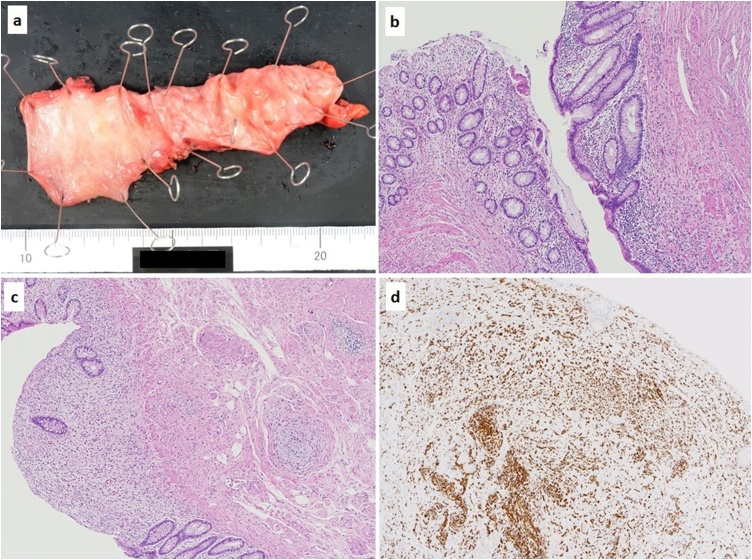
The resected specimen showed the appendiceal wall was fibrotic, enlargement, and thickness. There was no evidence of appendiceal rupture or serosal mucin extravasation (a). Histopathological examination showed atypical mucinous epithelial cells were lining by a single layer in the appendix (b). Multiple neurofibromas were revealed in the muscle layer, submucosa and mucosa of the appendix (c). Immunohistochemistry examination showed positive for S-100 (d).

A 62-year-old man with NF1 was scheduled for elective surgical treatment of an asymptomatic, enlarged and diffusely thickened appendix that remained after curative antimicrobial treatment of acute appendicitis because he hoped for an antimicrobial treatment 2 months ago. Physical examination revealed multiple neurofibromas and café-au-lait spots on the skin. He had no other co-morbidities other than having NF1. Laboratory analysis revealed hemoglobin, 15.9 g/dL; white blood cell count, 5.33 × 10^3^/μL; platelets, 17.9 × 10^4^/μL; serum total protein, 7.9 g/dL; serum albumin, 4.8 g/dL; total bilirubin, 0.8 mg/dL; aspartate aminotransferase, 27 IU/L; alanine aminotransferase, 21 IU/L; and lactic acid dehydrogenase, 173 IU/L; C-reactive protein, 0.073 mg/dL. The serum levels of tumor markers were normal, including carcinoembryonic antigen, 3.2 ng/ml and carbohydrate antigen 19–9, 7.1 U/mL. Contrast-enhanced computed tomography (CT) demonstrated an enlarged and diffusely thickened appendix (Fig. 1a, b). Colonoscopy showed thickened appendiceal mucosa projecting to the cecum without evidence of abscess or jellylike liquid (Fig. 2). A sample of the appendiceal mucosa was obtained by colonoscopy and pathologically revealed to be benign. The patient was preoperatively diagnosed with treated acute appendicitis with chronic appendiceal inflammation versus appendiceal neoplasms. Laparoscopic cecectomy was performed. The resected specimen revealed a thick, enlarged and fibrotic appendiceal wall. There was no evidence of appendiceal rupture or serosal mucin extravasation (Fig. 3a). Histopathological examination showed a single layer of atypical mucinous epithelial cells lining the appendix (Fig. 3b). Multiple neurofibromas were observed in the muscle layer, submucosa and mucosa of the appendix (Fig. 3c). Micro plexiform neurofibromas were observed in the neuroplexus of the appendix. Immunohistochemical examination showed positive staining for S-100 (Fig. 3d). Pathologically, the patient was diagnosed with appendiceal neurofibroma of NF1 with LAMNs. His postoperative course was unremarkable. He was discharged on post-operative day 3 and remained in good heath 7 months after surgery.

## 3 Discussion

NF1, also known as von Recklinghausen’s disease, is the most common autosomal dominant single-gene neurodevelopmental disorder, with an incidence of 1:2700 [5]. Gastrointestinal involvement has been reported in 10–25% of patients with NF1. Gastrointestinal neurofibromas usually occur in groups of multiple lesions, though solitary lesions have been reported. Lesions can be found, in order of frequency, in the jejunum, stomach, ileum, duodenum and colon. However, appendiceal neurofibromas in NF1 are extremely rare. To the best of our knowledge, only 7 cases [[6], [7], [8], [9], [10], [11], [12]] have been reported in the English literature (Table 1). Of these, 4 cases were diagnosed preoperatively as appendicitis.

**Table 1:** Reported cases of appendiceal neurofibromas.

No.	Author	Year	Age	Gender	Main symptom	Preoperative diagnosis	Surgeical procedure	Size (cm)
1	Merck and Kindblom [8]	1975	24	M	Abdominal pain	Appendicitis	Appendectomy	NA
2	Olsen [9]	1987	24	M	Abdominal pain	NA	Appendectomy	7 × 3
3	Samuel et al. [10]	1997	19	M	Abdominal pain	Appendicitis	Appendectomy	3 × 7×8
4	Rosenberg et al. [11]	2006	33	F	Asymptom	Finding unexpectedly in cesarean section	Appendectomy	12
5	Agaimy et al. [12]	2010	45	M	NA	NA	Appendectomy	0.3
6	Guo et al. [6]	2014	62	F	Abdominal pain	A giant thick-walled tubular mass	Right hemicolectomy	9 × 7
7	Ozaki et al. [7]	2015	51	M	Abdominal pain	Appendicitis	Appendectomy	3.5 × 2.5 × 2.5
8	Present case	2018	62	F	Asymptom	Cured appendicitis	Cecectomy	1.7 × 7

NA: not available.

Gastrointestinal neurofibromas are often asymptomatic. However, when the lesions grow in size, they may present as constipation, abdominal pain, palpable abdominal masses or bowel obstruction [13]. Neurofibromas are benign neoplasms consisting of the cells and tissues that cover nerves. However, there is a risk of malignant transformation, particularly in individuals over 40 years old [6]. Patients with NF1 have an 8–12% lifetime risk of developing malignant peripheral nerve sheath tumors (MPNSTs), a term designated by the World Health Organization in 2002 to replace the previous terms “malignant schwannoma”, “malignant neurilemmoma”, “neurogenic sarcoma”, and “neurofibrosarcoma” [14]. MPNSTs have a poor prognosis [15]. Neurofibromas were recently found to be precursor lesions for MPNSTs [15]. Therefore, rapid enlargement of neurofibromas should alert practitioners to the possibility of malignant transformation [6].

Surgical resection is the standard treatment for appendiceal neurofibromas and is aimed at improving symptoms, preventing complications and avoiding the risk of malignant transformation [16]. However, the best surgical procedure (appendectomy alone, cecectomy, ileocecal resection, or right hemicolectomy with or without regional lymph node dissection) for patients with appendiceal neurofibromas remains controversial. In the present case, the patient underwent laparoscopic cecectomy rather than laparoscopic appendectomy due to obtain the negative resection margin because he was suspected to have appendiceal neoplasm during surgery, for example LAMNs. It is difficult to diagnosis appendiceal neoplasms preoperatively. The patient would have had to undergo a two-stage ileocecal resection with or without regional lymph node dissection for a pathological diagnosis of mucinous adenocarcinoma or positive resection margin of the LAMNs. The most appropriate surgical procedure for appendiceal neurofibromas might be cecectomy, as the root of the appendix has the potential for malignant transformation and progression to MPNSTs. The surgical indication for patients without symptoms remains controversial. Many patients diagnosed with appendicitis preoperatively undergo appendectomy alone, even if they were pathologically diagnosed with appendiceal neurofibromas. Other groups have supported a two-stage ileocecal resection with regional lymph node dissection if patients are pathologically diagnosed with a malignant tumor or positive resection margins.

LAMNs are characterized by low-grade cytologic atypia and the absence of destructive invasion. These tumors have the potential for peritoneal spread giving rise to pseudomyxoma peritonei [17]. Thus, LAMNs are regarded as low-grade adenocarcinomas according to the 2010 World Health Organization classification [18]. To the best of our knowledge, this is the first report of appendiceal neurofibromas in NF1 with LAMNs in the English literature.

Surgical resection is the standard treatment for patients with LAMNs. However, the most appropriate surgical procedure for patients with LAMNs remains controversial. Generally, tumor involvement of a surgical margin is an indication for additional surgery. However, Arnason et al. reported that in patients with appendiceal LAMNs without discharge of mucin or exposure of the appendiceal serosa, involvement of the appendectomy margin by either neoplastic epithelium or acellular mucin was not associated with disease recurrence or peritoneal dissemination [19]. Therefore, in the present case, additional treatment for LAMNs was not necessary.

There are no guidelines for the optimal management strategy of appendiceal neurofibromas and LAMNs. Thus, the most appropriate surgical procedure for patients with appendiceal neurofibromas or LAMNs is unknown. At the very least, it is important to obtain negative resection margins. A larger number of long-term follow-up patients with appendiceal neurofibromas and LAMNs are required to establish optimal, evidence-based treatment.

## 4 Conclusions

We report herein a rare case of appendiceal neurofibroma with LAMN in NF1 patient. Patients with NF1 may have gastrointestinal tract involvement such as appendiceal neurofibromas. These cases are often preoperatively diagnosed as appendicitis. Therefore, it is necessary to suspect appendiceal neurofibromas in NF1 patient who have symptoms of appendicitis.

## Conflicts of interest

The authors declare that they have no conflicts of interest.

## Funding source

The authors declare that this study was not funded externally.

## Ethical approval

The study such as this case report was exempted from ethical approval by the Institutional Review Board of Hiroshima City Asa Citizens Hospital.

## Consent

When obtaining informed consent for surgical procedures, general consent for publication and presentation was obtained from the patient.

## Authors’ contributions

TK drafted the manuscript. TK and KO reviewed and edited the manuscript. TK, KO, TK, MY, NT, MK, AN, YA, MS, and MM participated in the care of the patients. MK and HM provided the histopathological examination and diagnosis. TK, JH, MF, HM, and NH participated in critical revision of the manuscript. All authors read and approved the final manuscript.

## Registration of research studies

This is case report not research study.

## Guarantor

Koichi Oishi.

## Provenance and peer review

Not commissioned, externally peer reviewed.
